# Long-term study (1987–2023) on the distribution of ^137^Cs in soil following the Chernobyl nuclear accident: a comparison of temporal migration measurements and compartment model predictions

**DOI:** 10.1093/rpd/ncad241

**Published:** 2023-09-11

**Authors:** Ioannis Kaissas, Alexandros Clouvas, Marios Postatziis, Stelios Xanthos, Michalakis Omirou

**Affiliations:** Nuclear Technology Laboratory, Department of Electrical and Computer Engineering, Aristotle University of Thessaloniki, GR-54124 Thessaloniki, Greece; Nuclear Technology Laboratory, Department of Electrical and Computer Engineering, Aristotle University of Thessaloniki, GR-54124 Thessaloniki, Greece; Nuclear Technology Laboratory, Department of Electrical and Computer Engineering, Aristotle University of Thessaloniki, GR-54124 Thessaloniki, Greece; Industrial Engineering and Management Department, International Hellenic University, GR-57400 Thessaloniki, Greece; Nuclear Technology Laboratory, Department of Electrical and Computer Engineering, Aristotle University of Thessaloniki, GR-54124 Thessaloniki, Greece

## Abstract

After the Chernobyl accident, a designated area of ~1000 m^2^ within the University farm of Aristotle University of Thessaloniki in Northern Greece was utilized as a test ground for radioecological measurements. The profile of ^137^Cs in the soil was monitored from 1987 to 2023, with soil samples collected in 5-cm-thick slices (layers) down to a depth of 30 cm. The mean total deposition of ^137^Cs in the area, backdated to the time of the Chernobyl accident, was determined to be 18.6 ± 1.8 kBq m^−2^ based on four follow-up profile measurements of ^137^Cs in the soil for the years 2022 and 2023. It is noteworthy that this value is similar the total deposition at the site, which was independently measured to be about 20 kBq m^−2^ during the first year after the Chernobyl accident. The fractional contribution of each soil layer (e.g., 0–5 cm, 5–10 cm, 10–15 cm, etc.) to the total deposition of ^137^Cs (0–30 cm) is presented and analyzed. A compartment model was utilized to forecast the temporal evolution of fractional contributions of the different soil layers to the total deposition of ^137^Cs (0–30 cm). In this model, each soil layer is represented as a separate compartment. The model assumes that the transfer rates between adjacent compartments are equal. The agreement between the measured fractional contributions and the model predictions suggests that the compartment model with equal transfer rates can capture the broad patterns of ^137^Cs migration within the soil layers over the long period of 1987–2023. However, the use of a second compartment model with increasing transfer rates between consecutive soil layers did not align with the observed outcomes. This indicates that diffusion may not be the primary migration mechanism over the 36-y period covered by our study.

## Introduction

The long-term external dose resulting from nuclear accidents, such as Chernobyl and Fukushima, primarily arises from the deposition of ^137^Cs in the environment. Accurate knowledge of the distribution of ^137^Cs deposited in the soil is crucial for reliable assessments of the external dose^(1, 2)^ and for understanding the potential uptake of this radionuclide by plants through root systems. Vertical migration of ^137^Cs in the soil has been a subject of extensive research^(3, 4)^ because of its importance in assessing environmental impacts and potential risks. The radioecological studies conducted following the Chernobyl accident have provided valuable insights and experience that could aid in the planning and interpretation of similar studies in Japan following the Fukushima accident. By leveraging the knowledge gained from the Chernobyl studies, researchers could enhance the effectiveness and efficiency of radioecological investigations in Fukushima.

After the Chernobyl accident, a designated area of ~1000 m^2^ within the University farm of Aristotle University of Thessaloniki in Northern Greece was utilized by the Nuclear Technology Laboratory as a test ground for radioecological measurements^(5, 6, 7)^. In the present study, follow-up profile measurements of ^137^Cs in the soil were conducted for the years 2022 and 2023 within the designated area. The measurements of the present study were performed ~10 y after the last measurements^(7)^ taken at the site, which spanned from 1987 to 2012. To analyze the data, the time evolution of the profile of ^137^Cs in soil was compared against the predictions of a simple compartment model for the entire period from 1987 to 2023.

## Materials and methods

The profile of ^137^Cs in the soil of the designated area was monitored from 1987 to 2023. Soil samples of undisturbed soil were collected in 5-cm-thick slices (layers) down to a depth of 30 cm. The soil samples were stored in plastic bags, air dried, (smashed to particles with dimensions lower than 2 mm) and used for ^137^Cs counting. The ^137^Cs activity of each soil layer was measured by standard gamma-spectroscopy. During the long study period spanning from 1987 to 2023, two high purity Germanium detectors were utilized. The first detector, a Canberra p-type coaxial HPGe of 20% relative efficiency, was in use from 1987 to 1999. Subsequently, the second detector, a Eurisys p-type coaxial HPGe of 50% relative efficiency, was employed from 2000 to 2023. The commercial shielding for each detector was provided by Canberra and Eurisys, respectively. Each gamma spectroscopy measurement had a duration of ~24 h. The combined standard uncertainty, with a coverage factor k = 1, for the ^137^Cs mass activity values (Bq/kg) was about 10%.

The soil characteristics are presented in Table 1

**Table 1:** Soil characteristics.

pH	8
Percent clay	22
Percent silt	37
Percent sand	41
Percent organic matter	1.28
Exchangeable potassium	0.48 meq 100 g^−1^
Total potassium	800 mg 100 g^−1^
CEC	22.28 meq 100 g^−1^
Percent CaCO_3_	1.8

Throughout the 36-y measurement period, no agricultural activities took place in the field. The sole human intervention consisted of cutting the surface grasses without disrupting the underlying soil. It is important to mention that one of the authors of the present study actively participated in all the measurements conducted at the site throughout this extended period from 1987 to 2023.

The results presented in this work provide information on the fractional contribution of each soil layer to the total deposition of ^137^Cs. The fractional contribution, denoted as R_z_, represents the ratio of the ^137^Cs mass activity in a specific layer to the total ^137^Cs mass activity in the entire deposition profile (0–30 cm). The fractional contribution R_z_ is simply given by: 


(1) \begin{equation*} {R}_z=\frac{A_z}{T} \end{equation*}

where A_z_ is the ^137^Cs mass activity (Bq/kg) in the soil layer z-5 to *z* with *z* = 5, 10, 15, 20, 25, 30 corresponding to the respective layers (0–5 cm, 5–10 cm, 10–15 cm, 15–20 cm, 20–25 cm, 25–30 cm) and T is the total in Bq/kg (Equation (2)). It should be noted that T is the sum of A_z_ with *z* = 5, 10, 15, 20, 25, 30 and not the ^137^Cs mass activity (Bq/kg) of the soil layer 0-30 cm. 


(2) \begin{equation*} T={A}_5+{A}_{10}+{A}_{15}+{A}_{20}+{A}_{25}+{A}_{30} \end{equation*}

The ratio of the activities of ^137^Cs and ^134^Cs (backdated to May 1986) the first years after the accident when the latter was measurable^(5)^ was found to be ~2 in all layers. Taking into account, that the same ratio 2 was recorded in air filters immediately after the arrival in Greece of the radioactive plume and that ^134^Cs in soil, is only because of the Chernobyl accident, we can suppose that practically all ^137^Cs in soil is because of the Chernobyl accident, i.e. nuclear weapon tests fallout is negligible in Greece.

## Results and discussion

The mean total deposition of ^137^Cs in the area, calculated retrospectively at the time of the Chernobyl accident, has been determined to be 18.6 ± 1.8 kBq m^−2^. This determination is based on four follow-up profile measurements of ^137^Cs in the soil conducted during the years 2022 and 2023. Significantly, this value closely resembles the total deposition observed at the site, which was independently measured^(5)^ to be ~20 kBq m^−2^ during the immediate year following the Chernobyl accident.

**Figure 1:**
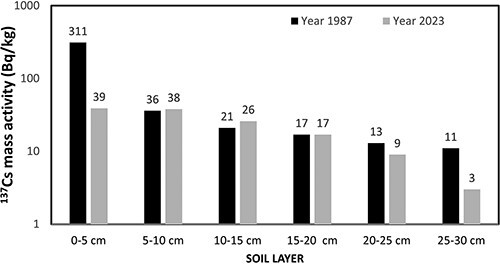
^**137**^Cs mass activity (Bq/kg) in different soil layers measured in 1987 and 2023.

**Figure 2:**
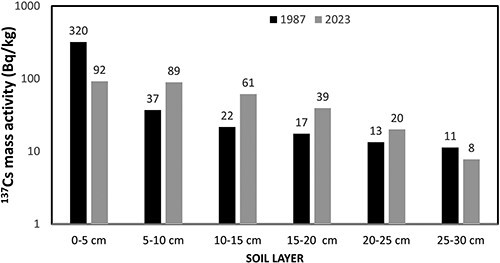
^**137**^Cs mass activity (Bq/kg) in different soil layers measured in 1987 and 2023. The results are backdated at the time of Chernobyl accident.

Figure 1 illustrates the ^137^Cs mass activity (Bq/kg) in the soil layers (0–5 cm, 5–10 cm, 10–15 cm, 15–20 cm, 20–25 cm, and 25–30 cm). These measurements represent the first and last ^137^Cs profiles taken at the site. The initial measurement was conducted in July 1987, ~1.2 y after the Chernobyl accident, whereas the final measurement was taken in April 2023, ~37 y after the Chernobyl accident. Figure 2 presents the same results as Figure 1, but they have been backdated at the time of the Chernobyl accident.

Knowing the soil’s density is 1.3 g/cm^3^, one can easily deduce the total deposition of ^137^Cs in kBq/m^2^ from the values presented in Figure 2. From the first and last profiles measured at the site, a total ^137^Cs deposition (backdated at the time of Chernobyl accident) of 27.3 ± 3 kBq m^−2^ and 20.2 ± 2 kBq m^−2^, respectively, was deduced. The agreement between these two values is considered satisfactory, especially given the extensive time span of 36 y between the two measurements.

**Figure 3:**
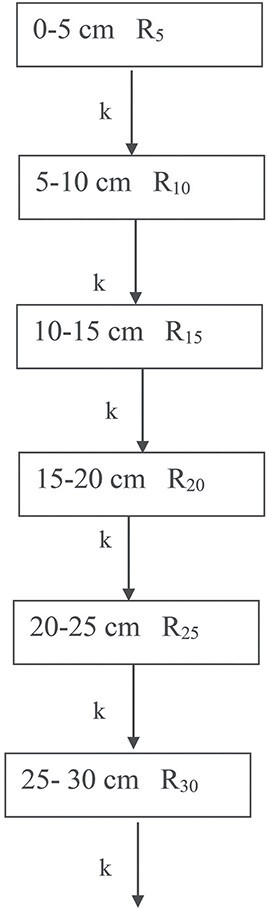
Schematic representation of the compartment model. R_5,_ R_10,_ R_15,_ R_20_, R_25,_ R_30_ are defined by Equation (1) and k are the transfer rates in y^−1^ between the compartments.

In the long-term evolution study of ^137^Cs distribution in soil over a period of ~36 y, we were able to investigate the time dependence of the specific ratios (Equation (1)), denoted as R_5_, R_10_, R_15_, R_20_, R_25_ and R_30_. Before showing the results is presented a simple compartment model (Figure 3) which is used to simulate the distribution of ^137^Cs in the soil over time. In the compartment model, layers of soil are represented by compartments. In our simple model, six soil layers are considered.

The differential equations describing the flow of ^137^Cs through the compartments are: 


(3) \begin{equation*} \frac{dR_5}{dt}=-k\ {R}_5 \end{equation*}

(4) \begin{equation*} \frac{dR_{10}}{dt}=k\ {R}_5-k\ {R}_{10} \end{equation*}

(5) \begin{equation*} \frac{dR_{15}}{dt}=k\ {R}_{10}-k\ {R}_{15} \end{equation*}

(6) \begin{equation*} \frac{dR_{20}}{dt}=k\ {R}_{15}-k\ {R}_{20} \end{equation*}

(7) \begin{equation*} \frac{dR_{25}}{dt}=k\ {R}_{20}-k\ {R}_{25} \end{equation*}

(8) \begin{equation*} \frac{dR_{30}}{dt}=k\ {R}_{25}-k\ {R}_{30} \end{equation*}

R_5,_ R_10,_ R_15,_ R_20_, R_25,_ R_30_ are defined by Equation (1) and *k* are the transfer rates in y^−1^ between the compartments. We remind that as R_z_ are ratios (Equation (1)), there is no need to take into account the decrease of ^137^Cs because of radioactive decay. The initial conditions for the fractional contributions R_z = 5, 10, 15, 20, 25, 30_ at the time of the Chernobyl accident (t = 0) are as follows: R_5_ = 0.71, R_10_ = 0.11,R_15_ = 0.06, R_20_ = 0.05, R_25_ = 0.04, R_30_ = 0.02. These values were deduced from the first ^137^Cs distributions performed in the area ^(5)^. For deposition by heavy rainfall, as it was the case in Greece during the Chernobyl accident, radionuclides percolate with rainwater and vertical migration in soil can be very fast during the early stage ^(3)^. This is the reason that only a year after the Chernobyl accident ^137^Cs could be found at even the depth of 30 cm. The solution of the differential equations is as follows:


(9) \begin{equation*} {R}_5(t)={R}_5\left(t=0\right){e}^{- kt} \end{equation*}

(10) \begin{equation*} {R}_{10}(t)=\left\{\left.\left(k\ {R}_5\left(t=0\right)t\right)+{R}_{10}\left(t=0\right)\right\}\right.{e}^{- kt} \end{equation*}

(11) \begin{align*} {R}_{15}(t)=&\left\{\left({k}^2{R}_5\left(t=0\right)\frac{t^2}{2}\right)+\left(k\ {R}_{10}\left(t=0\right)t\right)\right.\nonumber\\& +\,{R}_{15}\left(t=0\right)\Bigg\}{e}^{- kt} \end{align*}

(12) \begin{align*} {R}_{20}(t)=&\left\{\left({k}^3{R}_5\left(t=0\right)\frac{t^3}{6}\right)+\left({k}^2{R}_{10}\left(t=0\right)\frac{t^2}{2}\right)\right.\nonumber\\&+\left(k\ {R}_{15}\left(t=0\right)t\right)+{R}_{20}\left(t=0\right)\Bigg\}{e}^{- kt} \end{align*}

(13) \begin{align*} {R}_{25}(t)=&\left\{\left({k}^4{R}_5\left(t=0\right)\frac{t^4}{24}\right)+\left({k}^3{R}_{10}\left(t=0\right)\frac{t^3}{6}\right)\right.\nonumber\\&+\left({k}^2\ {R}_{15}\left(t=0\right)\frac{t^2}{2}\right)+\left(k\ {R}_{20}\left(t=0\right)t\right)\nonumber\\&+{R}_{25}\left(t=0\right)\Bigg\}{e}^{- kt} \end{align*}

(14) \begin{align*} {R}_{30}(t)=&\left\{\left({k}^5{R}_5\left(t=0\right)\frac{t^5}{120}\right)+\left({k}^4{R}_{10}\left(t=0\right)\frac{t^4}{24}\right)\right.\nonumber\\&+\left({k}^3{R}_{15}\left(t=0\right)\frac{t^3}{6}\right)+\left({k}^2{R}_{20}\left(t=0\right)\frac{t^2}{2}\right)\nonumber\\&+\left(k\ {R}_{25}\left(t=0\right)t\right)+{R}_{30}\left(t=0\right)\Bigg\}{e}^{- kt} \end{align*}

Equations (9–14) suggest that the factor k is the only free parameter in the model being used to describe the time evolution of different soil layers, specifically R_5_ (t), R_10_ (t), R_15_ (t), R_20_ (t), R_25_ (t) and R_30_ (t). It is acknowledged ^(8)^ that the value of k is not constant between consecutive soil layers and increases with soil depth, in case where diffusion is the important mechanism of migration. However, it is important to note that using too many free parameters can lead to overfitting, where a model excessively matches the data but fails to generalize well to new observations. To avoid this, the scope of our work is to investigate whether the use of only one free parameter (i.e. k is considered constant) can effectively describe the time evolution of the different soil layers R_z = 5, 10, 15, 20, 25, 30_.

**Figure 4:**
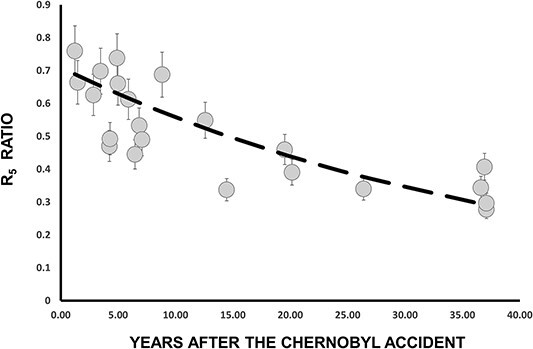
Time dependence of the R_5_ ratio. The dashed line is the best exponential function passing through the experimental values, taking into account that at the time of Chernobyl accident (t = 0) R_5_ = 0.71.

In Figure 4 is presented the time evolution of the R_5_ ratio. Despite the scattering of the values, one can clearly observe a decrease over the years of the fractional contribution of the 0–5 cm layer. The exponential function passing through the data (Figure 4) was determined using MATLAB’s ^(9)^ curve fitting tool, deducing the value of the k factor as *k* = 0.024 ± 0.006 y^−1^. This factor is quite similar (k = 0.027 y^−1^) to the one measured ^(7)^ in 2012, 10 y ago. From the k factor the mean residence time of ^137^Cs in the layer 0–5 cm (*t* = 41, 7 y) was deduced, and from that we can estimate the mean vertical migration velocity *v* = 0.12 cm y^−1^. The values obtained from these calculations are deemed reasonable and fall within the range of values reported in other studies (Almgren and Isaksson^(3)^, and references there in).

**Figure 5:**
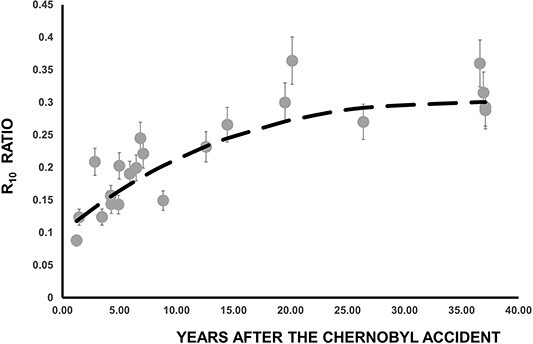
Time dependence of the R_10_ ratio. In dashed line are the results of Equation (10).

In Figure 5 is presented the time evolution of R_10_ ratio. As expected, an increase of R_10_ with time is observed. The interesting aspect is that the dashed line in Figure 5 does not represent a curve fitted to the experimental data points. Instead, it corresponds to the analytical predictions generated by the compartment model (Equation (10)). A relative good agreement between experimental and calculated R_10_ (t) values is observed. In addition it should be noted that 37 y after the Chernobyl accident the R_10_ (*t* = 37) values are quite similar to R_5_ (*t* = 37) values. The first year after the Chernobyl accident the R_10_ (*t* = 1) were about seven times smaller than R_5_ (*t* = 1) values. From Equation (10) is calculated that the maximum of R_10_ will be achieved in few years from now, about 40 y after the Chernobyl accident. After that, R_10_ will decrease slowly over time.

**Figure 6:**
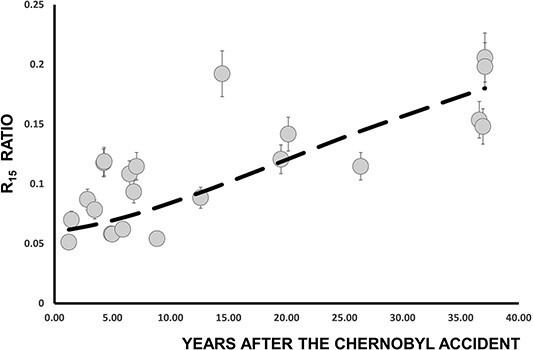
Time dependence of the R_15_ ratio. In dashed line are the results of Equation (11).

In Figure 6 is presented the time evolution of R_15_ ratio. As expected, an increase of R_15_ with time is observed. Again, as Figure 5, the dashed line in Figure 6 does not represent a curve fitted to the experimental data points. Instead, it corresponds to the predictions generated by the compartment model (Equation (11)). A relative good agreement between experimental and calculated R_15_ (t) values is observed.

**Figure 7:**
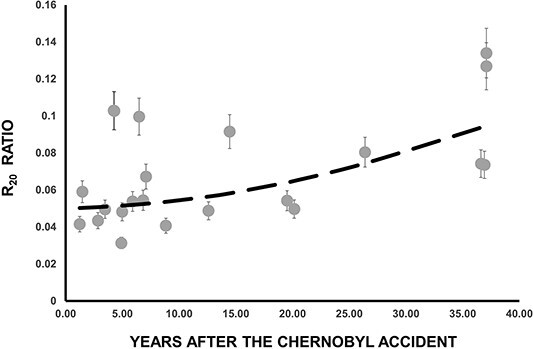
Time dependence of the R_20_ ratio. In dashed line are the results of Equation (12).

**Figure 8:**
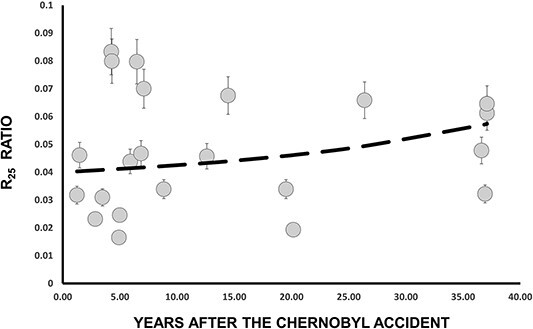
Time dependence of the R_25_ ratio. In dashed line are the results of Equation (13).

**Figure 9:**
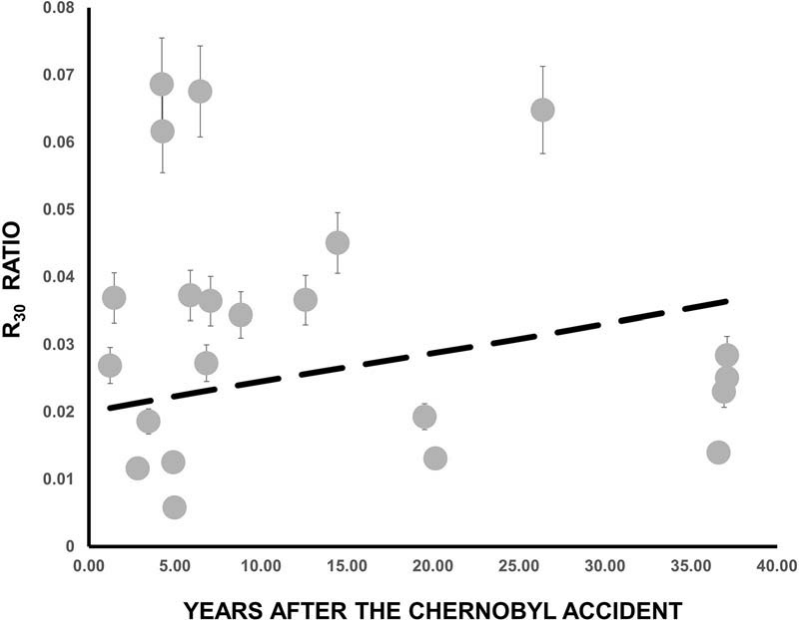
Time dependence of the R_30_ ratio. In dashed line are the results of Equation (14).

In Figure 7 is presented the time evolution of R_20_ ratio. In dashed line are the results of Equation (12). A satisfactory agreement between experimental and calculated R_20_ (t) values is observed. Figures 8 and 9 illustrate the time evolution of R_25_ and R_30_ ratios, respectively. In these figures, the dashed lines represent the results obtained from Equations (13 and 14). However, it is evident from these figures that drawing any definitive conclusions is challenging because of the scattering of the experimental data points.

**Figure 10:**
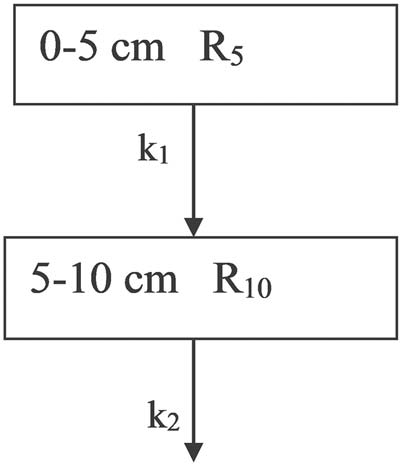
The schematic representation of the modified compartment model. k_1_, k_2_ are the transfer rates in y^−1^ between the compartments.

Overall, it is encouraging to observe a relative good agreement between the long-term time evolution of R_10,_R_15,_ R_20_ ratios with the predictions of the compartment model. This suggests that the compartment model employed in this study can provide a satisfactory description of the observed migration of ^137^Cs in soil over a period of 36 y. As mentioned previously, in case where diffusion is the important migration mechanism, the transfer rates k between the compartments are not the same, but increase with consecutive soil layers^(8)^ (compartments). To test this argument, we used the same model as before (Figure 3) but with k rates increasing between consecutive soil layers. In Figure 10 is shown the modified model for the first two compartments.

The differential equations describing the flow of ^137^Cs through the first two compartments are:


(15) \begin{equation*} \frac{dR_5}{dt}=-{k}_1\ {R}_5 \end{equation*}

(16) \begin{equation*} \frac{dR_{10}}{dt}={k}_1\ {R}_5-{k}_2\ {R}_{10} \end{equation*}

The transfer rates between these compartments, denoted as *k*_1_ and *k*_2_, are not the same as previously. Specifically, *k*_2_ is >*k*_1._ The initial conditions of R_5_ and R_10_ at *t* = 0 (the time of the Chernobyl accident) are R_5_ = 0.71 and R_10_ = 0.11, respectively.

The solution of the differential equations is as follows:


 (17) \begin{equation*} {R}_5(t)={R}_5\left(t=0\right){e}^{-{k}_1t} \end{equation*}

(18) \begin{align*} {R}_{10}(t)=&\left(\frac{k_1\ {R}_5\left(t=0\right)}{k_2-{k}_1}\right)\left({e}^{-{k}_1t}-{e}^{-{k}_2t}\right)\nonumber\\&+{R}_{10}\left(t=0\right){e}^{-{k}_2t} \end{align*}

**Figure 11:**
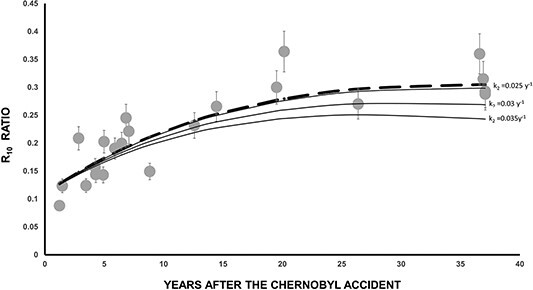
Time dependence of the R_10_ ratio. In dashed line are the results of Equation (10) with k = 0.024 y^−1^. In straight lines are the results of Equation (18) with different k_2_ values 4%, 25% and 46% greater than k_1_ = k = 0.024 y^−^.

Equation (17) is as Equation (9); therefore, k_1_ is the same as previously (*k* = 0.024y^−1^). In Figure 11 is presented the time dependence of the R_10_ ratio. In dashed line are the results of Equation (10) with *k* = 0.024 y^−1^. In straight lines are the results of Equation (18) with different k_2_ values >*k*_1_ = *k* = 0.024 y^−1^. It should be noted that Equation (18) is different from Equation (10). The results presented in Figure 11 demonstrate that the calculated results obtained from Equation (10), with *k* = 0.024 y^−1^, match the experimental results better than those obtained from Equation (18) with different k_2_ values 25%, 46% >*k*_1_ = *k* = 0.024 y^−1^. However, when *k*_2_ is slightly (4%) higher (k_2_ = 0.025 y^−1^) but approximately equal to *k*_1_ = *k* = 0.024 y^−1^, the calculated results from Equation (18) align closely with the results from Equation (10). As a conclusion, the use of a compartment model with increasing transfer rates between consecutive soil layers do not align with the observed outcomes. This suggests that diffusion may not be the primary migration mechanism over the long-term period of our study, which spans 36 y.

## Conclusions

In this work is presented a long-term study (1987–2023) of ^137^Cs distribution in soil because of the Chernobyl nuclear accident. Vertical migration of ^137^Cs in soil is a very slow process. The mean vertical migration velocity is estimated at 0.12 cm y^−1^. Based on the initial and most recent profiles taken at the site, 1 and 37 y following the Chernobyl nuclear accident, the deposition of ^137^Cs (backdated at the time of the Chernobyl nuclear accident) was determined to be 27.3 ± 3 kBq m^−2^ and 20.2 ± 2 kBq m^−2^, respectively. Notably, the agreement between these two results is deemed satisfactory, particularly considering the substantial 36-y gap between the two measurements. In the long-term evolution study of ^137^Cs distribution in soil over a period of ~36 y, we were able to investigate the time dependence of the specific ratios, denoted R_5_, R_10_, R_15_, R_20_, R_25_ and R_30_. The results are compared with model predictions where layers of soil are represented by compartments. Only one free parameter was used, i.e. the transfer rate between two consecutive compartments was the same for all compartments. Using too many free parameters can lead to overfitting, where a model excessively matches the data but fails to generalize well to new observations. A relative good agreement between the long-term time evolution of R_10,_R_15,_ R_20_ ratios with the predictions of the compartment model is observed. All of them (R_10,_R_15,_ R_20_) increase with time. The maximum of R_10_ will be achieved in few years from now, about 40 y after the Chernobyl accident. After that, R_10_ will decrease slowly over time. For the comparison between time evolution of R_25_ and R_30_ ratios with model predictions, drawing any definitive conclusions is challenging because of the scattering of the experimental data points. Overall, a relatively good agreement between the long-term time evolution of R_z_ ratios with the predictions of the compartment model is observed. This suggests that the compartment model employed in this study is capable of providing a satisfactory description of the observed migration of ^137^Cs in soil over a long period (1987–2023). On the other hand, the use of a second compartment model with increasing transfer rates between consecutive soil layers did not align with the observed outcomes. This suggests that diffusion may not be the primary migration mechanism over the long-term period of our study, which spans 36 y. Perhaps other migration mechanisms, apart from diffusion, play a significant role in the migration of ^137^Cs in the soil. It is possible that factors such as advection or other transport processes are more influential in the long-term migration patterns.

## Data Availability

The authors do not wish to publish the data of the study as their collection will continue and may be used enhanced in future studies.
